# Supplementary material for: Pivotal Role of Ubiquitin Carboxyl-Terminal Hydrolase L1 (UCHL1) in Uterine Leiomyoma
Source: Biomolecules. 2023 Jan 18;13(2):193. doi: 10.3390/biom13020193 (PMC9953523; doi:10.3390/biom13020193)
Supplement: Supplementary file 1 [file biomolecules-13-00193-s001.zip › Figure_S1-1973923R2.pdf]

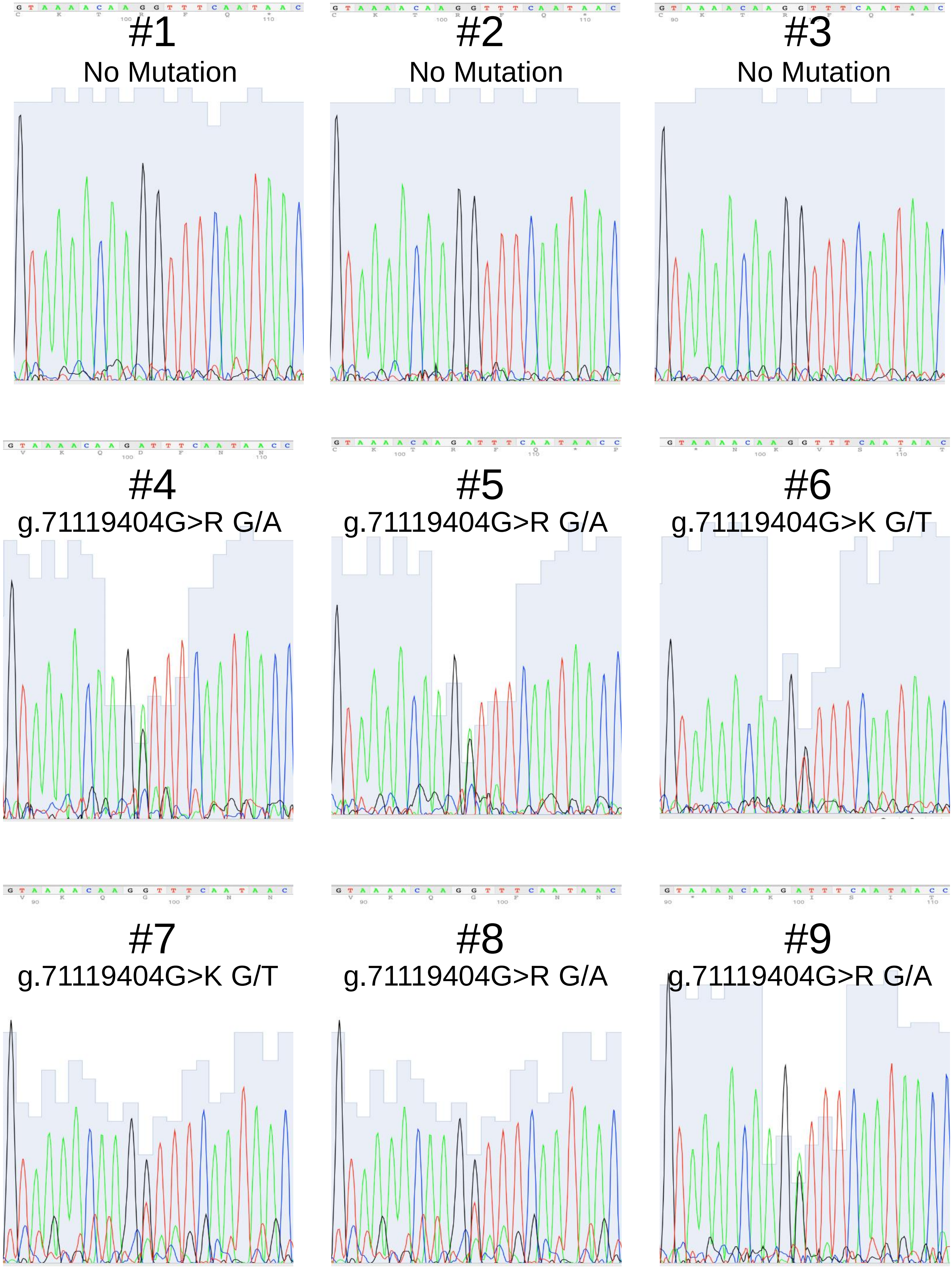
#1
No Mutation
#2
No Mutation
#3
No Mutation
#4
g.71119404G>R G/A
#5
g.71119404G>R G/A
#6
g.71119404G>K G/T
#7
g.71119404G>K G/T
#8
g.71119404G>R G/A
#9
g.71119404G>R G/A

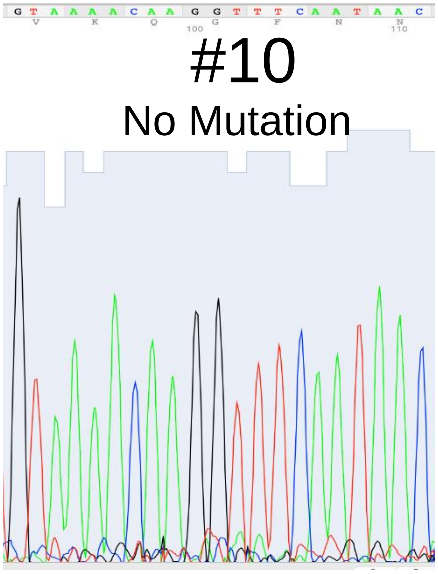

#10
No Mutation

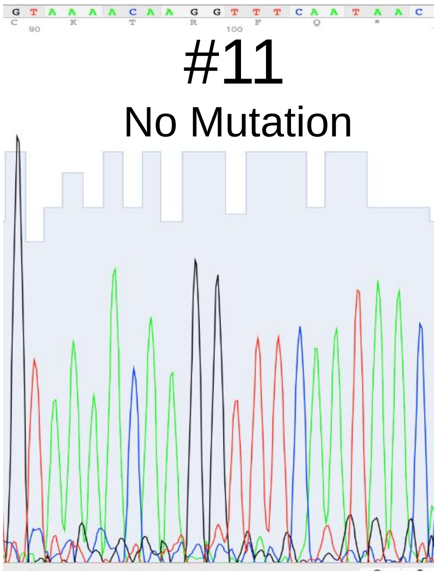

#11
No Mutation

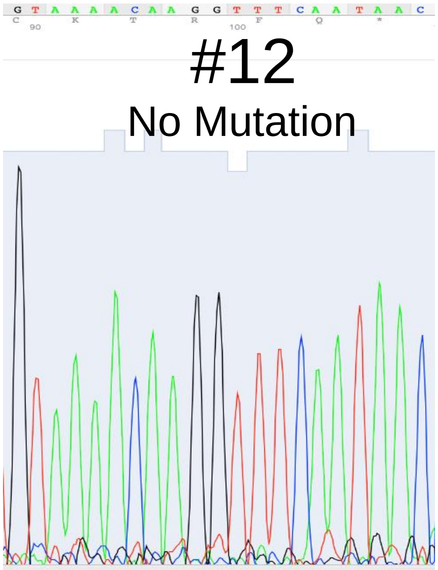

#12
No Mutation

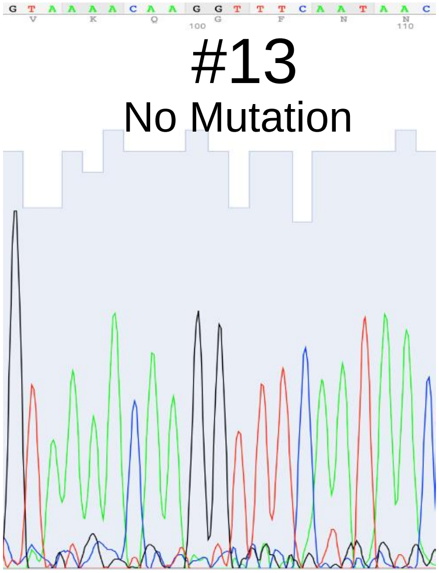

#13
No Mutation

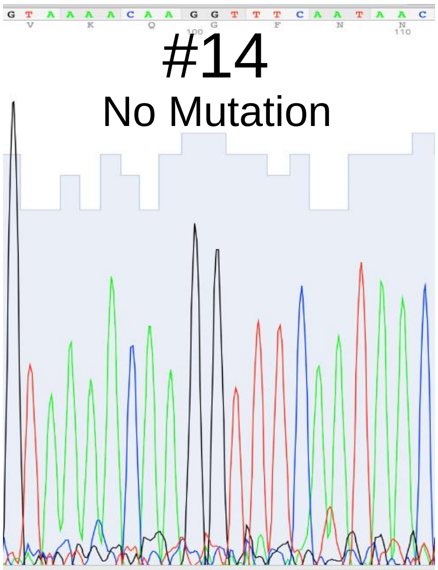

#14
No Mutation

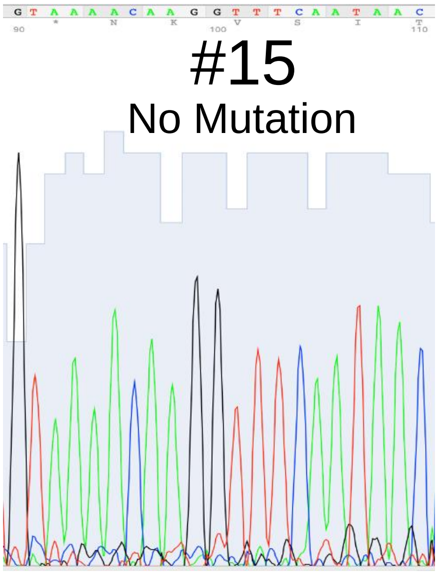

#15
No Mutation

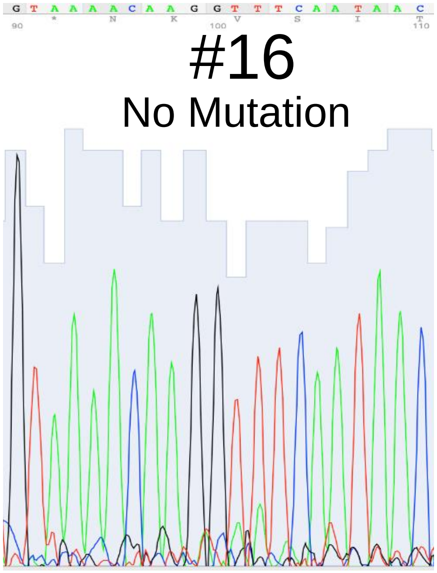

#16
No Mutation

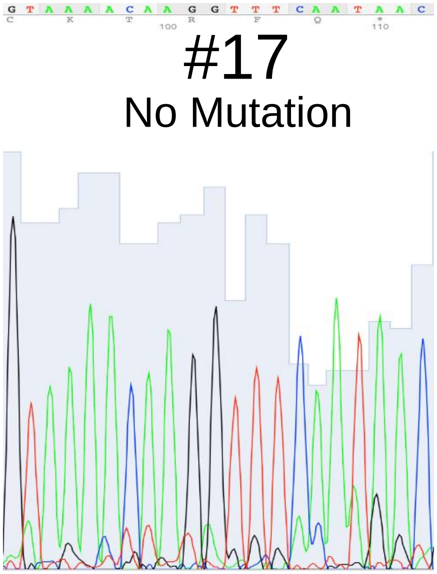

#17
No Mutation
