# Supplementary figures and images for: Pivotal Role of Ubiquitin Carboxyl-Terminal Hydrolase L1 (UCHL1) in Uterine Leiomyoma
Source: Biomolecules. 2023 Jan 18;13(2):193. doi: 10.3390/biom13020193 (PMC9953523; doi:10.3390/biom13020193)

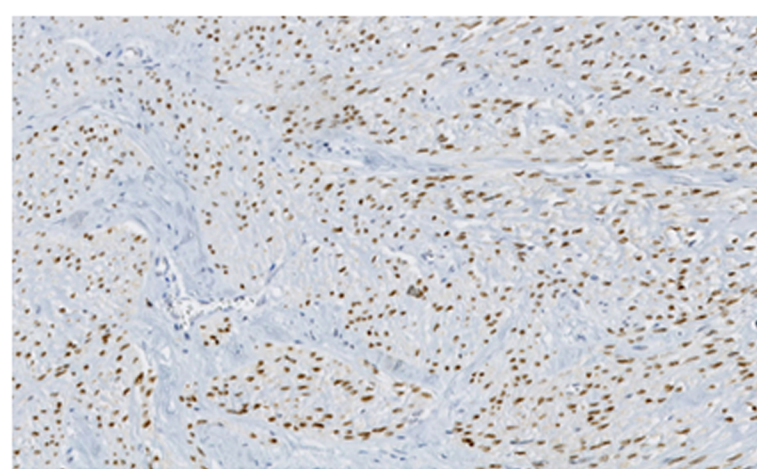

HMGA2++

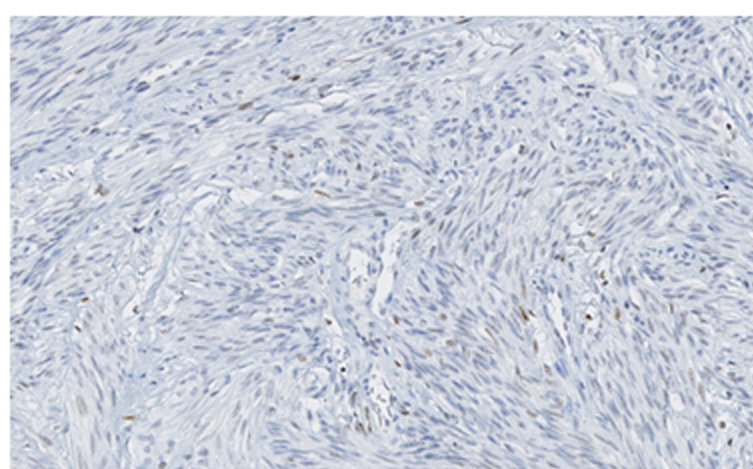

HMGA2+

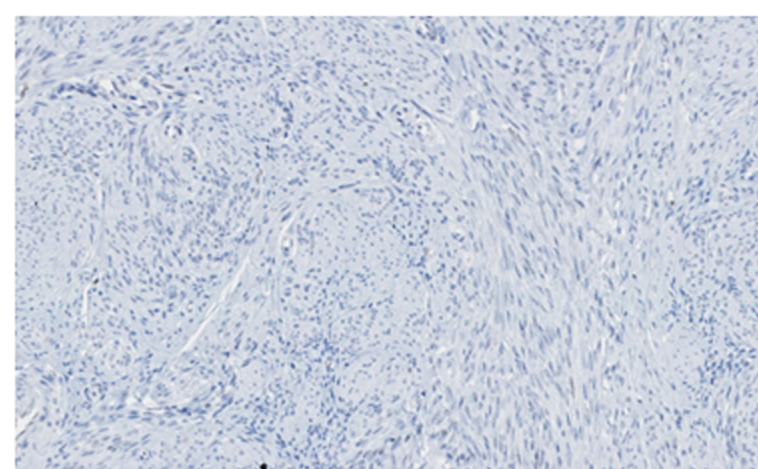

HMGA2-

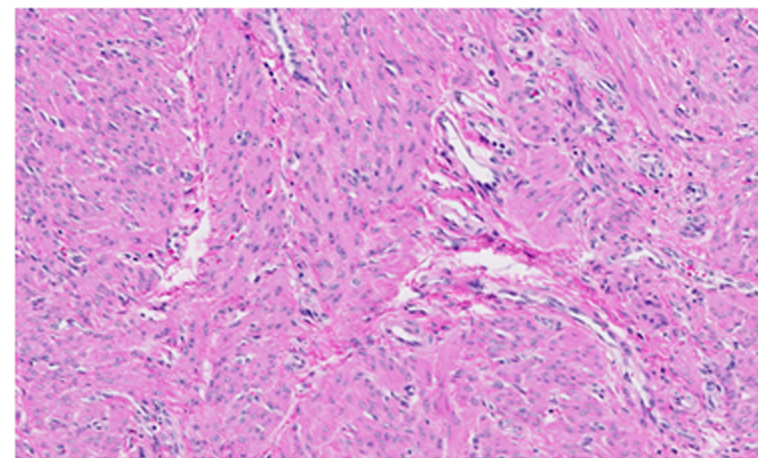

HE

Supplement: Supplementary file 1 [file biomolecules-13-00193-s001.zip › Figure S2-1973923R2.pdf]

10x

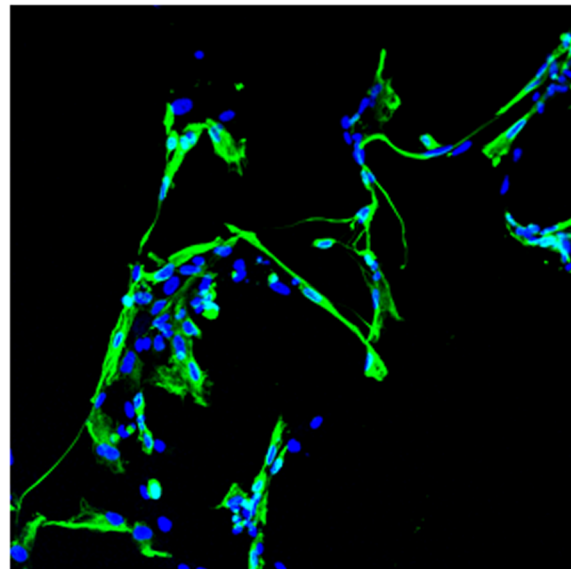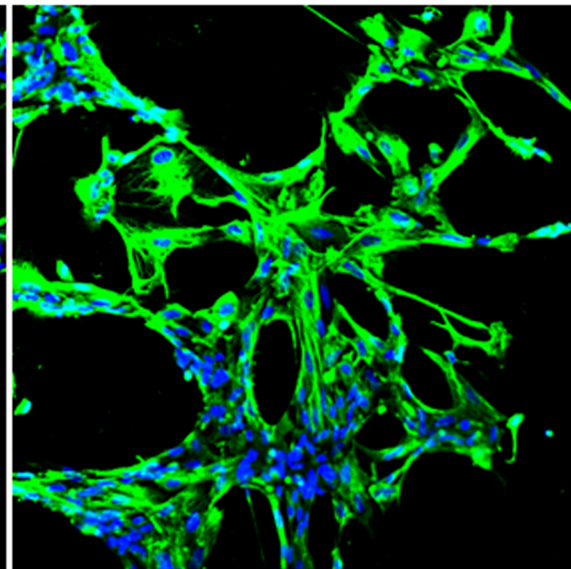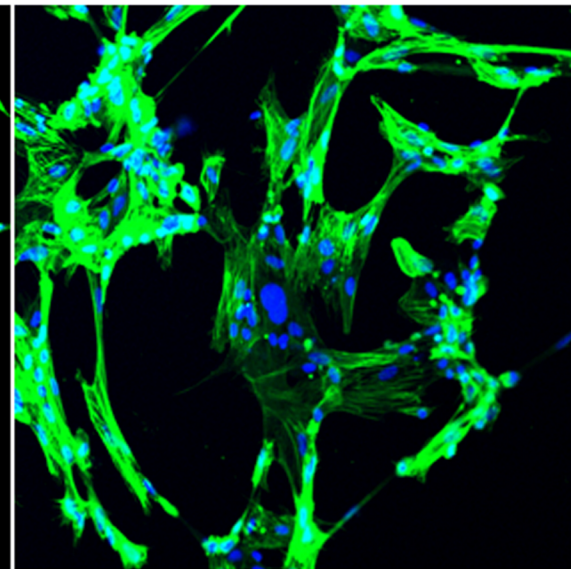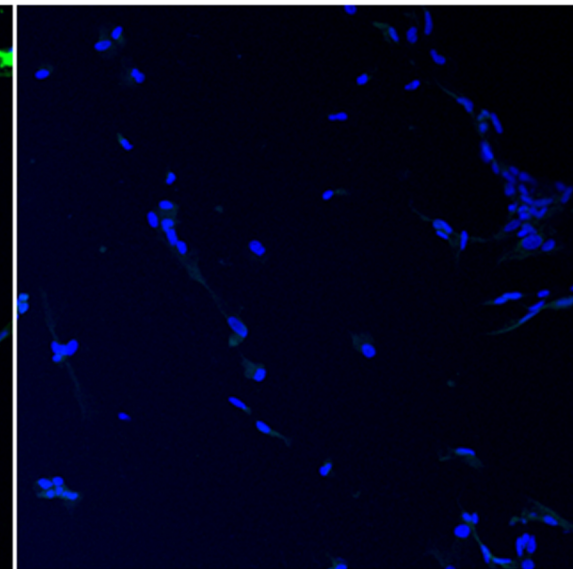

20x

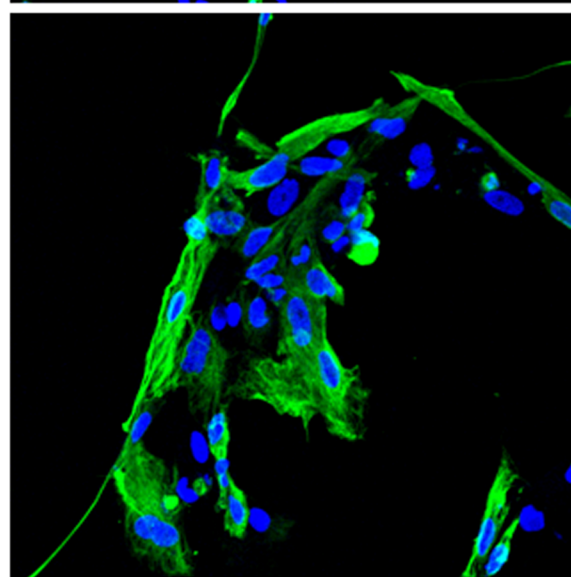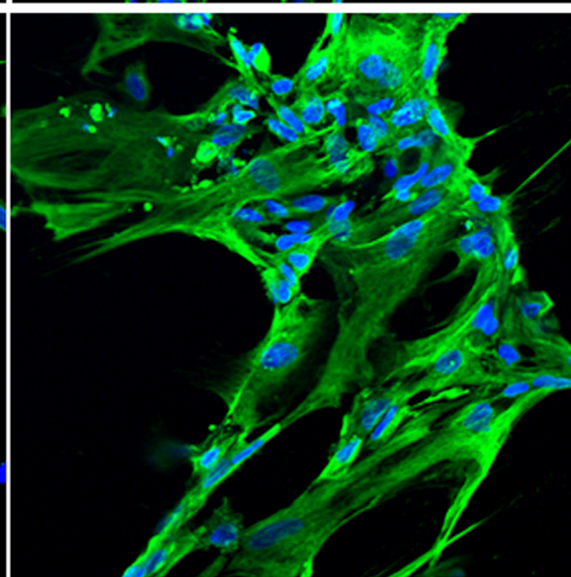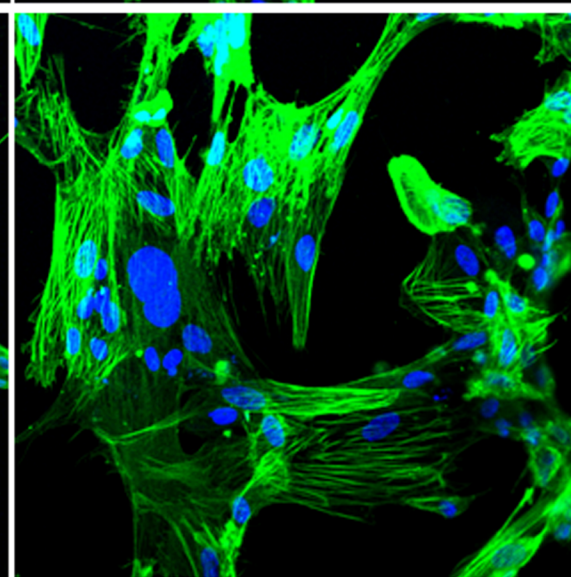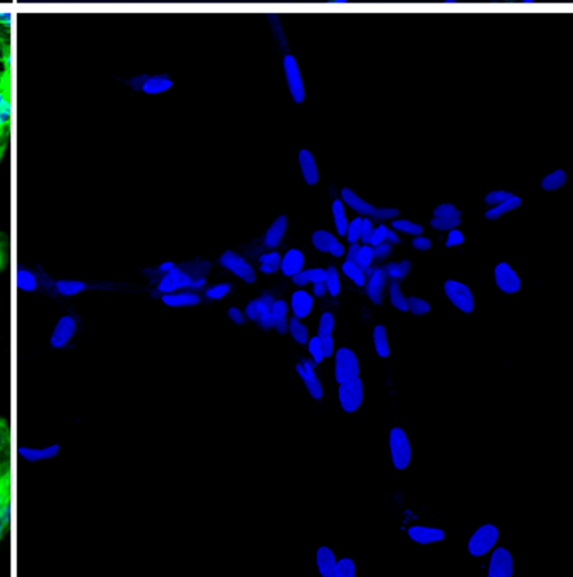

Desmin

Vimentin

ACTA2

Control

Supplement: Supplementary file 1 [file biomolecules-13-00193-s001.zip › Figure S3-1973923R2.pdf]
